# Metabolic Complications in Autoimmune and Autoinflammatory Diseases: Pathophysiology, Clinical Implications, and Therapeutic Perspectives

**DOI:** 10.3390/jcm15187199

**Published:** 2026-09-16

**Authors:** Emmanuel Andrès, Noel Lorenzo-Villalba, Nassim Dali-Youcef

**Affiliations:** 1 Service de Médecine Interne, Hôpital de Hautepierre, Hôpitaux Universitaires de Strasbourg, 67000 Strasbourg, France; 2 Laboratoire de Biochimie et Biologie Moléculaire, Pôle de Biologie, Hôpitaux Universitaires de Strasbourg, Nouvel Hôpital Civil, 67000 Strasbourg, France; 3 Institut de Génétique et de Biologie Moléculaire et Cellulaire (IGBMC), CNRS UMR 7104/INSERM U1258, Université de Strasbourg, 67400 Illkirch-Graffenstaden, France

**Keywords:** autoimmune diseases, autoinflammatory syndromes, metabolic syndrome, insulin resistance, cardiovascular disease, inflammation, antiphospholipid syndrome, glucocorticoids, atherosclerosis

## Abstract

**Background and objectives**: Autoimmune and autoinflammatory diseases extend far beyond their primary organ manifestations. Systemic inflammation in these disorders disrupts metabolic homeostasis, driving insulin resistance, dyslipidemia, accelerated atherosclerosis, and altered body composition. Metabolic syndrome (MetS) affects approximately 30% of patients with rheumatoid arthritis, with similar or higher prevalence in systemic lupus erythematosus, psoriasis, and antiphospholipid syndrome, contributing substantially to cardiovascular morbidity and mortality yet remaining underrecognized and undermanaged in routine clinical practice. This review summarizes the mechanisms linking chronic inflammation to metabolic dysfunction and the clinical epidemiology of metabolic complications across different autoimmune and autoinflammatory diseases, with attention to the metabolic effects of common immunomodulatory treatments. **Methods**: This narrative review was based on a literature search of PubMed/MEDLINE, Scopus, and Web of Science (publications available up to March 2026), using search terms covering autoimmune and autoinflammatory diseases, metabolic syndrome, insulin resistance, dyslipidemia, cardiovascular risk, cytokines, adipokines, and immunomodulatory treatments. **Results**: proinflammatory cytokines, adipokines, and immune cell populations were found to mediate insulin resistance and dyslipidemia across diseases. Conventional disease-modifying antirheumatic drugs (DMARDs), glucocorticoids, and emerging targeted biologics can either ameliorate or worsen metabolic disturbances depending on the specific agent and disease context. **Conclusions**: Understanding the bidirectional inflammation–metabolism relationship, together with treatment-related metabolic effects, is essential for comprehensive disease management and may identify novel therapeutic targets to reduce cardiovascular burden in this population.

## 1. Introduction

Autoimmune and autoinflammatory diseases affect approximately 5–10% of the population in developed countries, representing a substantial and growing burden on healthcare systems worldwide. While these conditions are traditionally characterized by their organ-specific or systemic inflammatory manifestations, accumulating evidence over the past 2 decades has revealed that chronic inflammation exerts profound effects on metabolic homeostasis. A recent large prospective cohort study of 369,065 individuals from the UK Biobank documented 4901 incident rheumatoid arthritis (RA) cases over 12 years of follow-up, demonstrating that metabolic syndrome and its components—including elevated waist circumference, elevated triglycerides, and reduced HDL cholesterol—were independently associated with increased RA risk [1]. Similarly, patients with established autoimmune conditions exhibit significantly enhanced prevalence and/or incidence of metabolic syndrome (MetS) [2,3,4], type 2 diabetes mellitus mainly in RA and psoriasis [5,6,7], and cardiovascular disease substantially attributable to disease-intrinsic inflammatory mechanisms compared with the general population [8,9,10].

The recognition that inflammation is not merely a consequence of metabolic disease but an active driver of metabolic dysfunction has transformed our understanding of disease pathogenesis [11]. A comprehensive meta-analysis reported an overall pooled MetS prevalence of 30.65% (variability from 14.32% to 37.83%) in RA patients with a 1.44 overall pooled odds ratio of MetS in RA patients compared to healthy controls. There was heterogeneity in the prevalence across studies that was attributed to age and differences in MetS diagnosis criteria [12]. Hence, there is a need for routine screening of components of the MetS in RA patients for cardiovascular risk management in this population.

Proinflammatory cytokines such as tumor necrosis factor-alpha (TNFα), interleukin-6 (IL-6), and interleukin-1 beta (IL-1β) directly interfere with insulin-signaling pathways, promote hepatic glucose production, alter lipid metabolism, and contribute to endothelial dysfunction [13].

Recent mechanistic investigations have shown that RA CD4+ T cells demonstrate low mitochondrial performance and rewired cellular metabolism, with glutamine-dependent pathways predominating in the glucose-depleted synovial environment [14]. Consistently, glutamine metabolism, regulated primarily by glutaminase 1 (GLS1), contributes to the pathogenesis of several autoimmune diseases. In rheumatoid arthritis, GLS1 drives synoviocyte proliferation, as GLS1 inhibition or silencing suppresses cell growth, while RA patient-derived synoviocytes display reduced proliferation in glutamine-deprived milieu, but not in glucose-deprived conditions, suggesting that glutamine metabolism is important in RA pathogenesis [15]. In psoriasis, GLS1 promotes pathogenic IL-17A-producing Th17 cell differentiation [16,17]. In SLE, GLS1 inhibition reduces Th17 activity in preclinical models and in patient-derived T cells, while expression of the counter-regulatory enzyme GLS2 is decreased in SLE T cells, compounding disease through oxidative stress and impaired IL-2 production [17]. Collectively, GLS1 acts as a proinflammatory mediator across autoimmune conditions, whereas GLS2 appears protective.

The cardiovascular consequences of metabolic complications in autoimmune disease are not trivial. A 2023 systematic literature review and meta-analysis evaluating cardiovascular disease (CVD) in systemic lupus erythematosus (SLE) demonstrated that compared with adults without SLE, patients with SLE had statistically significantly higher relative risks of stroke (2.51 [2.03–3.10]), myocardial infarction (2.92 [2.45–3.48]), CVD (2.24 [1.94–2.59]), and hypertension (2.70 [1.48–4.92]) [18]. Traditional cardiovascular risk factors alone do not fully account for the excess cardiovascular risk observed in autoimmune diseases, with large-scale epidemiological data demonstrating that this risk persists independently after adjustment for established confounders; dysregulated cytokine signaling, particularly via IL-1β, TNFα, and IL-6, drives endothelial dysfunction and proatherogenic lipoprotein remodeling, thereby contributing directly to accelerated atherosclerosis and increased cardiovascular events. Understanding the pathophysiology linking chronic inflammation to metabolic dysfunction, along with recognition of treatment-associated metabolic effects, is therefore critical for comprehensive risk assessment and may reveal novel therapeutic targets to mitigate cardiovascular morbidity and mortality in this high-risk population.

### Literature Search and Selection

This narrative review was based on a literature search of PubMed/MEDLINE, Scopus, and Web of Science for publications available up to March 2026. Search terms included combinations of “autoimmune disease,” “autoinflammatory disease,” “rheumatoid arthritis,” “systemic lupus erythematosus,” “antiphospholipid syndrome,” “psoriasis,” “psoriatic arthritis,” “inflammatory bowel disease,” “metabolic syndrome,” “insulin resistance,” “dyslipidemia,” “cardiovascular risk,” “atherosclerosis,” “thrombosis,” “cytokines,” “adipokines,” “immunometabolism,” and “glucocorticoids.” Priority was given to original mechanistic and clinical studies, randomized controlled trials, large observational cohorts, and systematic reviews or meta-analyses. Recent authoritative reviews were used to provide context for broad or emerging areas. Reference lists of selected publications were also screened for additional relevant studies. Because of the narrative design and broad clinical scope of the review, study selection was based on relevance, methodological quality, and contribution to the mechanistic or clinical questions addressed, rather than on a formal systematic-review protocol.

## 2. Pathophysiological Mechanisms Linking Inflammation to Metabolic Dysfunction

### 2.1. Cytokine-Mediated Insulin Resistance and Metabolic Dysfunction

**Figure 1:**
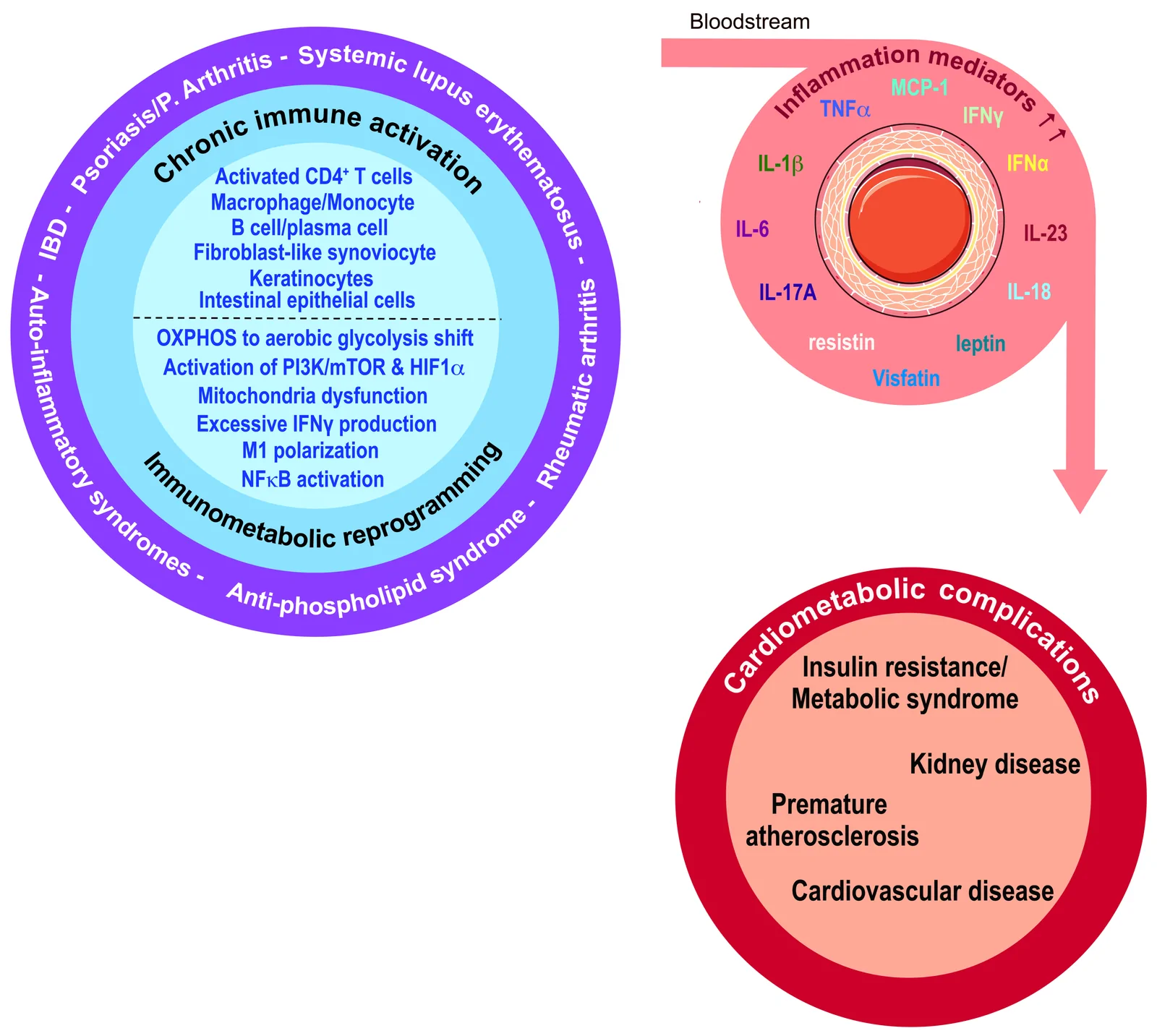
Metabolic complications of autoimmune and autoinflammatory diseases. Chronic immune activation in autoimmune and autoinflammatory diseases (RA, SLE, psoriasis/PsA, APS, IBD, and autoinflammatory syndromes) drives immunometabolic reprogramming within activated immune and tissue-resident cells, characterized by a shift from oxidative phosphorylation to aerobic glycolysis, PI3K/mTOR–HIF1α activation, mitochondrial dysfunction, and M1/NF-κB-driven polarization. This reprogramming fuels the systemic release of proinflammatory cytokines, interferons, and adipokines (TNFα, IL-6, IL-1β, IL-17A, IL-23, IL-18, MCP-1, IFN-α/γ, leptin, resistin, visfatin) into the bloodstream. Circulating at elevated levels, these mediators propagate local tissue inflammation into systemic cardiometabolic dysfunction, manifesting as insulin resistance/metabolic syndrome, premature atherosclerosis, cardiovascular disease, and kidney disease.

Insulin resistance represents a central metabolic abnormality in autoimmune and autoinflammatory diseases, characterized by reduced cellular responsiveness to insulin in skeletal muscle, adipose tissue, and liver. Multiple inflammatory mediators interfere with insulin signaling at various levels of the cascade (Figure 1).

**TNFα**, one of the most extensively studied proinflammatory cytokines, activates serine kinases, including c-Jun N-terminal kinase (JNK) and inhibitor of κB kinase (IKK), which phosphorylate insulin receptor substrate-1 (IRS-1) on serine residues (pS307) rather than the tyrosine residues necessary for normal insulin signaling. This serine phosphorylation inhibits the interaction between IRS-1 and the insulin receptor, effectively blocking downstream signal transduction through the phosphatidylinositol 3-kinase (PI3K)/Akt pathway that mediates glucose uptake and glycogen synthesis [19]. TNFα levels are significantly increased in rheumatoid factor (RF)-positive and anti-cyclic citrullinated peptide (CCP)-positive patients compared to patients who are negative for RF and anti-CCP antibodies. Moreover, TNFα levels are positively correlated with homeostatic model assessment index for insulin resistance (HOMA-IR) in RA patients [20]. Interestingly, anti-CCP-positive patients exhibit significantly higher Disease Activity Score in 28 joints (DAS28) and are more insulin resistant compared with anti-CCP-negative patients [20] (Table A1). In SLE patients, TNFα levels are more associated with dyslipidemia than with insulin resistance, with TNFα and triglycerides found to be independent determinants of disease activity [21,22] (Table A1). The causal role of TNFα in mediating metabolic abnormalities in psoriasis/PsA such as insulin resistance remains unclear and is often extrapolated from studies conducted in the context of obesity and metabolic inflammation. Nevertheless, one study established a positive correlation between circulating TNFα and HOMA-IR, and between disease activity—as measured by DAPSA score—and HOMA-IR in patients with psoriatic arthritis (PsA) (n = 32 versus n = 32 healthy controls) [23] (Table A1). Consistently, anti-TNF therapy has been shown to directly improve insulin sensitivity in nondiabetic patients with psoriasis [24]. In antiphospholipid syndrome (APS) patients, a direct correlation of TNFα with insulin resistance has not been firmly demonstrated. In the study of Rodrigues et al., although the percentage of MetS and HOMA-IR were significantly increased in APS patients compared to healthy controls, insulin resistance signal was attributed to leptin, PAI-1, and adiponectin dysregulation and TNFα was not measured in this study [25] (Table A1). However, TNFα levels were strongly associated with venous thrombosis in patients with APS [26] (Figure 1 and Table A1).

**IL-6**, another key cytokine elevated in many autoimmune conditions, promotes insulin resistance through distinct converging mechanisms. IL-6 induces expression of suppressor of cytokine signaling 3 (SOCS3) via JAK/STAT signaling, which directly inhibits insulin receptor signaling through SOCS3 binding to Tyr960 of the insulin receptor, thereby preventing IRS1 recruitment and tyrosine phosphorylation in hepatocytes [27,28]. IL-6 can also cause insulin resistance through SOCS3-dependent ubiquitination and proteasomal degradation of IRS proteins [29]. Chung et al. have reported a significant positive correlation between circulating IL-6 levels and HOMA-IR index in RA patients, but not for SLE patients [30] (Table A1). In multivariate models, the major contributing factors to HOMA-IR were BMI for SLE, and IL-6 and TNFα levels in RA patients [30]. RA is the disease in which the IL-6/insulin resistance axis is best characterized, both mechanistically and clinically. The evidence base for IL-6 inhibitor-mediated improvement of insulin resistance is essentially confined to RA. Intravenous administration of the anti-IL-6 receptor antibody tocilizumab produced a rapid and significant improvement in HOMA-IR and insulin sensitivity (QUICKI) in nondiabetic RA patients, supporting a direct causal role for IL-6 in RA-associated insulin resistance [31]. These findings were corroborated in a study demonstrating a significant decrease in insulin levels and HOMA-IR, along with increased adiponectin levels compared with baseline values, following 3 months of tocilizumab therapy in non-diabetic patients with RA [32] (Table A1). Of note, in RA, SOCS3 is overexpressed in macrophages and synovial fibroblasts in response to excessive IL-6-mediated STAT3 activation, yet this overexpression is insufficient to control the excess inflammatory signal, resulting in persistent metabolic dysregulation [33]. In addition, it was found that RA and SLE patients had more proinflammatory dysfunctional HDL particles (associated with atherosclerosis and CVD) compared to controls [34] (Table A1). Similarly to TNFα, increased IL-6 levels were associated with thrombosis in primary APS patients compared with individuals with no history of thrombosis [26] (Figure 1 and Table A1).

**MCP-1/CCL2.** Macrophage chemoattractant protein-1 (MCP-1)/CC-Chemokine ligand 2 (CCL2) links local tissue inflammation to systemic metabolic dysfunction through its role in recruiting monocytes into adipose tissue [35]. In rheumatoid arthritis specifically, a case-control study of 138 RA patients and 138 controls found that CCL2 serum levels and expression of its receptor, CCR2, were directly associated with insulin resistance status, with the highest adiposity and metabolic burden observed in RA patients with insulin resistance compared with RA patients without insulin resistance or with controls [36] (Table A1 and Figure 1). This provides disease-specific evidence that the CCL2/CCR2 axis is a shared inflammatory pathway linking RA to metabolic dysfunction, consistent with the chemokine’s broadly recognized role in monocyte-driven inflammation. No approved biologic currently targets MCP-1/CCL2 directly in RA, and CCR2 antagonists remain investigational. No direct evidence of an association between MCP-1 and metabolic complications in other autoimmune diseases has been reported to date.

**IL-1β, IL-18 and the NLRP3 inflammasome.** IL-1β contributes to metabolic dysfunction through its effects on pancreatic β-cells and peripheral tissues. In addition to promoting insulin resistance by activating inflammatory signaling pathways (e.g., JNK/NF-κB) in peripheral tissues (e.g., adipocytes and liver), glucose-induced IL-1β production in β-cells triggers NF-κB activation followed by Fas-induced β-cell apoptosis, culminating in glucose-stimulated insulin secretion impairment [37]. This mechanism contributes to the transition from compensatory insulin resistance to overt diabetes mellitus.

The NLRP3 inflammasome, a multiprotein assembly complex comprising NLRP3, ASC, and procaspase-1 that processes IL-1β and IL-18 from their inactive precursors, has emerged as a critical mediator converting metabolic stress into sustained inflammation [38]. In MetS, metabolic danger signals, including saturated fatty acids [39], cholesterol crystals [40], and hyperglycemia-mediated ROS increase (through ROS-dependent thioredoxin-interacting protein (TXNIP) binding with NLRP3) [41], activate NLRP3 to promote caspase-1-dependent release of IL-1β and IL-18, and gasdermin D-dependent pyroptosis [42]. The resulting cytokine excess directly worsens insulin resistance, accelerates endothelial dysfunction, and promotes atherogenesis, establishing a pathological loop wherein metabolic alterations and inflammasome-induced inflammation amplify each other, which ultimately will converge into cardiovascular complications [43] (Figure 1).

In RA, synovial inflammasome activation is implicated as a driver of IL-1β and IL-18 release in synovial fluid compared to osteoarthritis patients, with significantly higher serum levels of both cytokines compared to healthy controls [44], whilst MetS confers a ~44% additional cardiovascular risk beyond RA inflammation alone [12]. Moreover, it was recently demonstrated that insulin resistance in RA patients was associated with higher circulating IL-1β levels and higher cumulative 28-Joint Disease Activity Score (DAS28), pointing to a contributing role of IL-1β in mediating insulin resistance in RA [45]. Moreover, IL-1β blockade with anakinra, but not TNFα inhibition, was shown to improve insulin resistance in RA patients with T2D [46] (Table A1). In SLE, NLRP3 overactivation sustains nephritis and accelerates atherosclerosis [47], while inflammasome-mediated activation of IL-18 results in endothelial progenitor cell (EPC) dysfunction in SLE patients, and neutralization of IL-18 in cultured EPC cells from SLE patients restores their capacity to differentiate into mature endothelial cells [48]. Moreover, IL-18 levels are associated with insulin resistance and cardiovascular risk factors such as hypertriglyceridemia, hyperhomocysteinemia and vascular stiffness in SLE patients [49,50]. However, although IL-18 was associated with atherosclerosis (positive correlation with carotid intima-media thickness) in RA patients, no correlation with HOMA-IR was found [51]. In APS, antiphospholipid antibody-driven endothelial dysfunction and oxidative stress, hallmarks of the disease, contribute to thrombo-inflammation in microvascular and macrovascular beds [52,53], with recent metabolomic data linking this state to dyslipidemia, glucose and amino acid metabolism dysregulations, and increased cardiovascular risk [54]. In psoriasis, variants in genes encoding components of the NLRP3 inflammasome complex are associated with an increased prevalence of atherosclerotic cardiovascular disease beyond traditional risk factors [55] (Table A1). A genomic score could be implemented in new algorithms for cardiovascular disease risk assessment. Clinical trials targeting NLRP3 pathways showed that IL-1β antagonism showed partial efficacy in refractory pustular psoriasis [56] but showed only modest benefit in active psoriatic arthritis [57], while anti-TNFα agents indirectly normalized inflammasome activity that may reduce cardiovascular risk [58]. Thus, IL-1β antagonists are not used as a first-line medicine in plaque psoriasis and PsA, where other cytokines are targeted.

In the autoinflammatory syndrome CAPS (Cryopyrin-associated periodic syndrome), gain-of-function mutations in NLRP3 cause constitutive activation of the NLRP3 inflammasome without the need for cell priming, resulting in increased cleavage of gasdermin D, increased release of IL-1β and IL-18, and pyroptosis, alongside a concurrent proinflammatory gene signature. Interestingly, NLRP3 variants in CAPS were shown to be associated in myeloid cells with immunometabolism dysregulation with disrupted lipid and amino acid pathways and impaired glycolysis, limiting IL-1β production, yet preserving a basal inflammatory status as a defense mechanism against pathogens [59] (Table A1). The translation to the clinic is provided by the CANTOS trial demonstrating that IL-1β blockade reduces major cardiovascular events [60] (Table A1).

Beyond the classical cytokines discussed above, other proinflammatory cytokines contribute to metabolic complications in autoimmune diseases with clinical implications across the disease spectrum.

**IL-17A/IL-23 axis**. IL-17A is an established proinflammatory cytokine not only in organ inflammation and autoimmune diseases, but accumulating evidence points to its contribution to vascular and metabolic complications in these diseases [61,62]. Indeed, anti-IL17A therapy improves cutaneous and articular manifestations, and favorably modulates systemic and vascular inflammation in psoriasis and PsA, though effects on cardiovascular endpoints and metabolic parameters remain limited and need further investigation [63,64,65] (Table A1). IL-23 acts upstream of IL-17 and is not required for initial induction of Th17 differentiation, but it is essential for the stabilization, expansion, and full effector competence of Th17 cells to secrete IL-17 and IL-22 [66,67]. It was recently demonstrated that psoriatic patients receiving biologics targeting inflammatory cytokines, including IL-17 and IL-23, display a significantly lower cardiovascular risk than those under oral nonbiologic antipsoriatic therapy [65] (Table A1). Moreover, IL-17 plays a dual role in adipocyte physiology. In preadipocytes, IL-17A restrains adipogenesis by inhibiting proadipogenic transcription factors (C/EBP-α, PPARγ, and KLF5) and inducing the antiadipogenic KLF2 and KLF3 [68]. In differentiated adipocytes, however, IL-17 impairs insulin-mediated glucose uptake and insulin sensitivity; accordingly, young IL-17-deficient mice show improved glucose tolerance and insulin sensitivity despite higher body weight on both low- and high-fat diets, indicating a protective effect of IL-17 against obesity that is lost in age-associated obesity [69]. Consistently, diet-induced IL-17A drives CDK5-dependent PPARγ phosphorylation at Ser273, altering obesity- and diabetes-associated gene expression, while suppressing the IL-17A axis prevents diet-induced obesity and its metabolic abnormalities, supporting a genuine contribution to diet-induced metabolic inflammation and insulin resistance [70]. Adipose IL-23 expression, meanwhile, correlates positively with inflammatory markers (TNFα and IL-1β), fasting glucose, HbA1c and HOMA-IR index, and negatively with adiponectin [71]. However, targeting the IL23/IL-17A axis has not translated into clinical improvement of metabolic parameters, particularly insulin resistance: in two psoriasis trials, secukinumab (anti-IL-17A) produced a null or worse HOMA-IR response than placebo [63,72]. A 1-year study of methotrexate, anti-IL17, anti-IL23, and anti-TNFα in psoriasis found that although all therapies significantly reduced PASI, patients with concomitant MetS improved less than those without—with BMI and leptin levels predicting delayed response—suggesting MetS may blunt treatment efficacy through compounded inflammatory and metabolic disturbances [73]. Anti-IL-17 produced the greatest sustained long-term improvements, and anti-IL-23 the fastest early response; both nonetheless improved metabolic parameters (decreased leptin, cholesterol, and triglycerides; increased HDL-C), though insulin resistance was not assessed in these patients [73]. A separate study similarly found anti-IL-17 and anti-IL-23 biologics improved both PASI and metabolic indicators (glucose, lipids, CRP, ESR, IL-6) compared with cyclosporine, with MetS again reducing efficacy, though less so and with a better long-term response in the anti-IL-23 group [74] (Table A1). Overall, these findings suggest that managing MetS could improve biologic efficacy in psoriasis, through the bidirectional relationship between inflammation and metabolism warrants further study in psoriasis and autoimmune diseases more broadly (Figure 1).

**Interferons.** Type I and type II interferons (IFNs) contribute to metabolic dysregulation in certain autoimmune and autoinflammatory diseases, though with disease-specific mechanisms. In SLE, the type I IFN signature tracks with disease activity, and elevated serum IFN-α (type I), together with IFN-γ (type II), independently associates with dyslipidemia and impaired β-cell function [75] (Table A1); mechanistically, chronic type I IFN signaling impairs mitochondrial gene expression and metabolic fitness in CD8+ T cells [76] and correlates with subclinical vascular damage and cardiovascular biomarkers independent of traditional Framingham risk factors [77] (Table A1). In primary antiphospholipid syndrome (APS), high type I IFN activity is associated with impaired endothelial progenitor function that is reversible with anti-Type I IFN antibody [78] (Table A1 and Figure 1), though no study has linked type I IFN activity to MetS components in APS as it has in SLE. In RA, serum IFN-α correlates with rheumatoid factor and anti-CCP positivity and with disease activity, whereas IFN-γ levels shows little association with clinical manifestations; neither IFN correlates with cardiovascular comorbidities such as lipid profiles, insulin resistance, or carotid ultrasound findings [79]. More broadly, IFN-γ depletes regulatory T cells in visceral and subcutaneous adipose tissue in human obesity, impairing adipocyte insulin sensitivity [80]; whether this specific IFN-γ–Treg–adipocyte axis operates in psoriasis/PsA or RA remains unclear, warranting further investigation.

### 2.2. Dyslipidemia and Lipid Paradox

Lipid metabolism is profoundly altered in autoimmune and autoinflammatory diseases, characterized by a pattern often termed “inflammatory dyslipidemia”. This lipid profile typically includes elevated triglycerides, reduced high-density lipoprotein cholesterol (HDL-C), and qualitative changes in lipoprotein composition and function. Indeed, lipid metabolism in RA is altered not only quantitatively but also qualitatively, with HDL particles losing their atheroprotective functions [81,82]. Paradoxically, total cholesterol and low-density lipoprotein cholesterol (LDL-C) levels are often reduced during periods of high disease activity, a phenomenon known as the “lipid paradox” [83]. This reduction in LDL-C does not indicate decreased cardiovascular risk. Rather, it reflects the impact of inflammatory mediators on hepatic lipoprotein synthesis and enhanced cholesterol uptake by activated immune cells and inflamed tissues.

Proinflammatory cytokines significantly alter lipoprotein metabolism at multiple levels. TNFα and IL-6 suppress expression of lipoprotein lipase (LPL), the enzyme responsible for hydrolysis of triglycerides in very-low-density lipoprotein (VLDL) and chylomicrons, leading to hypertriglyceridemia. Simultaneously, these cytokines stimulate hepatic de novo lipogenesis and VLDL secretion [84]. Inflammation also modifies the composition and function of HDL particles, reducing their cholesterol efflux capacity and antioxidant properties while increasing their content of proinflammatory proteins such as serum amyloid A (SAA) [85]. These dysfunctional proinflammatory HDL (piHDL) particles lose their atheroprotective effects and may paradoxically promote inflammation and atherogenesis. This was demonstrated in a cohort of RA patients in which piHDL was associated with active disease and altered protein cargo compared to anti-inflammatory, functionally preserved HDL (aiHDL) observed in RA patients with low disease activity and healthy controls [81]. Consistently, anti-TNFα therapy with infliximab was shown to improve HDL function in RA patients treated with methotrexate plus infliximab compared to those receiving methotrexate plus placebo, suggesting that the TNFα pathway plays an active role in modulating the vascular properties of HDL [86]. Similarly, psoriasis was shown to alter HDL composition and cholesterol efflux capacity with a shift towards a proinflammatory profile in HDL particles of psoriatic patients compared to those of healthy controls [87].

Furthermore, inflammation promotes oxidative modification of LDL particles [88], generating oxidized LDL (oxLDL) that is particularly proatherogenic [89]. OxLDL is preferentially taken up by macrophages through scavenger receptors (e.g., SR-A1, CD36 and LOX-1), leading to foam cell formation, a hallmark of early atherosclerotic lesions [89,90]. Interestingly, macrophages are not the only source of foam cells: oxLDL is also captured by vascular smooth muscle cells (VSMCs) through LOX-1, SR-A, CD36, and other scavenger receptors, causing VSMCs to re-differentiate into foam cells. VSMC-derived foam cells are thought to play an important role in plaque stability as their apoptosis promotes the formation and expansion of necrotic cores and macrophage infiltration [91]. It is worth noting that the role of inflammation in inducing LDL oxidation is not straightforward. Hence, oxLDL activates endothelial cells, promotes expression of adhesion molecules that allow monocyte chemotaxis, and triggers inflammatory responses that perpetuate a vicious cycle of chronic vascular inflammation [90]. Studies in patients with RA and psoriasis have demonstrated elevated levels of oxLDL that correlate with disease activity rather than atherosclerosis as determined by IMT measurements, suggesting that other causes of CVD such as plaque stability, rather than a general increase in atherosclerosis, may be more important to explain the increased CVD incidence in RA [92,93,94]. Finally, multiple clinical studies reported an increased oxidized LDL profile in patients with SLE compared with controls [95,96,97,98], and oxLDL levels were associated with arterial disease and renal manifestations [95].

## 3. Adipose Tissue Dysfunction and Adipokine Dysregulation

Adipose tissue has emerged as a key player in the intersection of inflammation and metabolism, functioning not merely as an energy storage depot but as an active endocrine and immunological organ [99,100]. In the context of chronic systemic low-grade inflammation, adipose tissue undergoes significant remodeling characterized by infiltration of proinflammatory immune cells, particularly M1-polarized macrophages and T helper 1 (Th1) cells [101,102]. Adipokines are strongly associated with MetS in RA, although they were not independently associated with clinical response to advanced therapies, highlighting the complex interplay between adipose tissue, inflammation, and treatment outcomes [103]. These infiltrating immune cells, along with activated adipocytes, secrete a range of proinflammatory cytokines and adipokines that contribute to both local and systemic metabolic dysfunction [101,102].

Adipokines, bioactive proteins secreted primarily by adipocytes, play crucial roles in regulating insulin sensitivity, energy homeostasis, and inflammation [104,105]. In certain autoimmune diseases, the adipokine profile is characteristically altered, with increased levels of proinflammatory adipokines such as leptin, resistin, and visfatin [106]. More recently, additional adipokines have been shown to play immunomodulatory roles in rheumatic joint diseases such as progranulin, chemerin, lipocalin-2, vaspin, omentin-1, and nesfatin [107]. Consistently, more than a decade ago, it was reported that in RA patients, adipokines, inflammation, insulin resistance, and carotid atherosclerosis are intricately linked, with specific adipokine patterns correlating with subclinical cardiovascular disease [108]. Adiponectin normally enhances insulin sensitivity through activation of AMP-activated protein kinase (AMPK) [109] and suppresses inflammatory responses through inhibition of nuclear factor-κB (NF-κB) signaling [110,111]. Adiponectin levels are inversely correlated with body mass index (BMI), waist circumference, and components of the MetS, independent of obesity [112], while lower adiponectin levels were demonstrated to predict the incidence of the MetS in a prospective study [113]. In a multivariate analysis, it was shown that obesity and diabetes are associated with low adiponectin levels and that hypoadiponectinemia was more closely related to the degree of insulin resistance and hyperinsulinemia rather than to the degree of adiposity and glucose intolerance in different ethnic groups [114] (Figure 1).

Adiponectin dysregulation in RA, SLE, and psoriatic arthritis (PsA) does not follow the pattern of hypoadiponectinemia characteristic of obesity-driven metabolic disease. Paradoxically, circulating adiponectin is elevated rather than reduced in RA and SLE, yet this hyperadiponectinemia does not appear to confer atheroprotection, with elevated levels associating positively with disease severity, radiographic progression, and cardiovascular risk rather than inversely, as would be expected from its canonical metabolic role [106,115]. This “adiponectin paradox” likely reflects tissue-specific signaling dysfunction and isoform heterogeneity—with adiponectin exhibiting context-dependent proinflammatory properties within the joint synovium and other inflamed tissues—rather than a straightforward loss of protective function [116,117]. Most importantly, it was demonstrated that adiponectin is able to promote the production of proinflammatory cytokines in peripheral blood mononuclear cells (PBMCs) and fibroblast-like synoviocytes (FLS) from non-inflamed individuals, supporting the possibility that adiponectin is involved in early phases of RA pathogenesis [118]. Consistently, in a longitudinal non-randomized study involving 3693 individuals from the Swedish Obese Subjects (SOS) followed for up to 29 years, high serum adiponectin at baseline was associated with increased risk of developing RA. Of interest, subjects with high adiponectin and CRP levels at baseline were particularly at risk of developing RA [119]. PsA patients had significantly higher serum levels of adiponectin than healthy controls (*p* < 0.05), with adiponectin being independently associated with PsA in logistic regression analysis (*p* = 0.001; OR = 3.1; 95% CI = 1.6–6.0), although no correlation with disease activity (DAPSA) was observed [120]. The pathophysiological significance of these results needs further clarification in other studies. By contrast, in psoriasis, adiponectin predominantly follows the obesity/metabolic pattern, i.e., negative correlation with BMI and decreased levels in psoriasis patients with MetS compared to patients without MetS, rather than an autoimmune inflammation-driven pattern as in RA or SLE. Psoriasis severity and antipsoriatic treatment had no relevant impact on adiponectin levels [121]. These findings were corroborated in a meta-analysis (n = 63 studies, 2876 psoriasis patients and 2237 healthy controls), demonstrating significantly lower adiponectin serum levels in psoriasis patients and increased levels of proinflammatory cytokines (IL-6, IL-18, and TNFα, among others) compared with controls [122]. Circulating levels of Visfatin, also named NAMPT (nicotinamide phosphoribosyl-transferase) and PBEF (pre-B-cell colony-enhancing factor), are consistently elevated in RA patients compared to healthy individuals and studies suggest that visfatin levels correlate with disease activity, with elevation potentially preceding clinical relapse symptoms [123,124]. However, in the ESPOIR prospective study (n = 791 patients), contrary to adiponectin, serum visfatin levels and radiographic RA progression were found to be unrelated [125], probably because visfatin is elevated in early diagnosed untreated RA patients [126] (Table A1). In this study, circulating visfatin levels were found to positively correlate with insulin resistance, cholesterol, triglycerides, LDL-C, and disease activity score [126]. Visfatin is not merely an elevated circulating adipokine in RA but is also produced intra-articularly by synoviocytes and induces MMP production and inflammatory cytokines, participating thereby in joint destruction [127]. Mechanistically, NAMPT expression is increased during inflammation and identified as a novel mediator of innate immunity. NAMPT expressed by Ly6C-high inflammatory monocytes promotes IL-6 production, which in turn drives Th17 cell expansion. APO866, a specific competitive inhibitor of NAMPT, effectively reduced arthritis severity with comparable activity to etanercept, and addition of NMN (the product of the NAMPT-catalyzed reaction) restored both intracellular NAD levels and TNFα/IL-6 production, thereby confirming the NAD dependence in the inflammatory process [128] (Table A1). The association of visfatin with SLE is controversial and appears to be context-dependent, reflecting a real heterogeneity driven by disease activity, study population, and assay variability. A meta-analysis (n = 11 studies) showed that overall visfatin-SLE association was not robust across all studies, yet stratification according to region showed a significantly increased visfatin level in SLE patients compared to controls in the Western and South American populations, but not in the Middle Eastern population [129]. Hence, a direct comparative study across rheumatic diseases found visfatin not elevated in SLE patients [130], which could be explained by the absence of disease activity stratification probably due to the small number of SLE patients (n = 26). However, in the study by Chung et al. (n = 109 SLE vs. 78 controls), significantly higher visfatin concentrations were reported in SLE patients compared to controls [131], findings corroborated by De Sanctis et al. [132]. More recently, Shaker et al. demonstrated increased serum visfatin levels in patients with active lupus nephritis compared with inactive lupus patients and healthy controls, while a robust positive correlation with disease activity index was documented [133]. However, unlike in RA patients, visfatin was not reported to correlate with metabolic complications (e.g., insulin resistance, dyslipidemia) in SLE or other autoimmune diseases.

Leptin, an adipokine often elevated in obesity and MetS, acts as a proinflammatory cytokine that promotes Th1 differentiation, stimulates macrophage-derived inflammatory mediators, and contributes to endothelial dysfunction [134]. As in insulin resistance, chronic hyperleptinemia can drive leptin resistance, sustaining this proinflammatory state despite adequate adipose reserves. This disruption of the pro/anti-inflammatory adipokine balance likely contributes to both metabolic dysfunction and accelerated atherosclerosis in autoimmune disease [134,135]. Among adipokines, leptin shows the most consistent link to metabolic dysfunction in autoimmune disease: in rheumatoid arthritis (RA), leptin correlated with HOMA-IR independent of BMI and inflammatory markers, while resistin and visfatin showed no such association [108,136] (Table A1). Plasma leptin and visfatin (but not resistin) were also elevated in RA versus controls [137]. In SLE, leptin levels significantly and independently correlated with insulin levels, triglycerides, BMI, corticosteroid levels, and SLE Disease Activity Index [138]. In psoriasis, a meta-analysis confirmed consistently elevated leptin and resistin alongside reduced adiponectin [139]. Patients with psoriasis had significantly higher levels of leptin, resistin, triglycerides, and total cholesterol, along with increased carotid artery intima-media thickness (cIMT). cIMT also correlated positively with these biomarkers, suggesting that psoriasis may independently contribute to the risk of subclinical atherosclerosis [140] (Table A1). Emerging evidence shows that in psoriasis patients, resistin levels were positively correlated with cholesterol and triglycerides and associated with T2D and MetS [141] (Table A1). In SLE, resistin has shown inconsistent results across studies. Baker et al. found that SLE patients had higher resistin levels than controls, with resistin correlating with inflammatory markers and renal disease, but not with insulin resistance or BMI [142]. In contrast, Almehed et al. found no difference in resistin levels between SLE patients and controls, although among SLE patients, resistin still correlated with markers of inflammation, renal function, and glucocorticoid dose [143]. In APS, leptin was positively associated with insulin resistance [25].

## 4. Endothelial Dysfunction and Accelerated Atherosclerosis

Endothelial dysfunction represents an early and crucial step in atherogenesis, characterized by impaired endothelium-dependent vasodilation, increased permeability, and a prothrombotic, proinflammatory phenotype [144]. In autoimmune and autoinflammatory diseases, endothelial dysfunction is driven by multiple inflammatory mediators, oxidative stress, and immune-mediated injury [145,146]. Proinflammatory cytokines, including TNFα, IL-1β, and IFN-γ, activate endothelial cells, inducing expression of adhesion molecules such as vascular cell adhesion molecule-1 (VCAM-1), intercellular adhesion molecule-1 (ICAM-1), and E-selectin, which facilitate recruitment of leukocytes to the arterial wall [147].

Inflammation also impairs endothelial nitric oxide (NO) bioavailability through multiple mechanisms. Proinflammatory cytokines suppress expression and activity of endothelial nitric oxide synthase (eNOS), while oxidative stress generated by activated leukocytes and dysfunctional mitochondria leads to rapid inactivation of NO through reaction with superoxide radicals, forming peroxynitrite. Reduced NO bioavailability not only impairs vasodilation but also diminishes the antiatherogenic properties of NO, which normally inhibits platelet aggregation, leukocyte adhesion, smooth muscle cell proliferation, and oxidative modification of lipoproteins [147,148].

Accelerated atherosclerosis observed in autoimmune diseases shares features with conventional atherosclerosis but also exhibits disease-specific characteristics. In addition to traditional risk factors and inflammatory mechanisms common to all autoimmune conditions, certain diseases involve specific pathogenic features that further promote vascular injury. For example, in SLE, antiphospholipid antibodies and anti-endothelial cell antibodies directly damage the endothelium and promote a proadhesive and proinflammatory phenotype, features that favor a prothrombotic state [149,150,151]. In RA, synovial inflammation may provide a sustained source of proinflammatory cytokines with systemic effects on vascular biology [152]. Studies using advanced imaging techniques, including positron emission tomography (PET) with fluorodeoxyglucose (FDG-PET), have demonstrated increased arterial wall inflammation in patients with active autoimmune disease, providing direct evidence of vascular involvement in these conditions [153,154,155].

## 5. Clinical Epidemiology of Metabolic, Atherosclerotic, and Thromboembolism Risks Across Autoimmune Diseases

For each disease below, metabolic, atherosclerotic, and thromboembolic risk are summarized in turn; the underlying cytokine-level mechanisms are detailed in the Pathophysiological Mechanisms section above and summarized by disease in Table A1, while therapeutic and monitoring implications are addressed collectively in Cardiovascular Risk Management Strategies below.

### 5.1. Rheumatoid Arthritis

Rheumatoid arthritis, affecting approximately 0.5–1% of adults worldwide, is perhaps the best-studied autoimmune condition with respect to metabolic complications.

Metabolic risk: Multiple large cohort studies and meta-analyses have consistently demonstrated increased prevalence of MetS in RA patients, with a pooled global prevalence of 30.3% [156]. A 2023 cross-sectional analysis of 65 RA patients found that those with MetS exhibited significantly higher disease activity scores (DAS28), elevated C-reactive protein (CRP), and increased synovial hypertrophy and vascularization on ultrasound compared to RA patients without MetS [1,156]. Insulin resistance is present in up to 40% of RA patients, even in the absence of obesity or overt diabetes, and is independently associated with disease activity markers. A 2025 UK Biobank study of over 500,000 individuals confirmed that RA, along with psoriatic arthritis and axial spondyloarthritis, showed significantly elevated odds of MetS even after adjustment for age, sex, CRP, and smoking status [157]. RA patients often exhibit a paradoxical body composition termed “rheumatoid cachexia,” characterized by loss of lean muscle mass with preserved or increased fat mass, which may further exacerbate insulin resistance and metabolic dysfunction [158].

Atherosclerotic risk: The cardiovascular burden in RA is substantial, with studies reporting a 1.5-to-2-fold increased risk of myocardial infarction, stroke, and cardiovascular death compared with the general population [159]. Importantly, this excess risk is not fully explained by traditional cardiovascular risk factors, suggesting significant contributions from RA-specific inflammatory mechanisms [160]. The inflammatory dyslipidemia characteristic of RA, with elevated triglycerides, low HDL-C [161], and dysfunctional HDL particles, contributes to accelerated atherosclerosis [81,82].

Thromboembolic risk: Venous thromboembolism (VTE) has been associated with RA. A recent meta-analysis of 14 observational studies found RA patients had a significantly increased risk of VTE (pooled risk ratio 1.57, 95% CI 1.41–1.76), deep vein thrombosis (RR 1.58, 95% CI 1.26–1.97), and pulmonary embolism (RR 1.57, 95% CI 1.30–1.88) compared with the general population, consistent across cohort, case-control, and cross-sectional study designs [162]. An earlier, smaller meta-analysis of nine observational studies reported a similarly elevated pooled VTE risk ratio of 1.96 (95% CI 1.81–2.11), reinforcing the association [163]. Mechanistic pathways underlying these associations are summarized in Table A1; therapeutic and monitoring approaches are discussed in Cardiovascular Risk Management Strategies.

### 5.2. Systemic Lupus Erythematosus

Systemic lupus erythematosus presents a particularly severe metabolic phenotype, with studies demonstrating MetS prevalence of 30–50% in SLE cohorts.

Metabolic risk: A 2015 landmark study found that in SLE patients, MetS was not only a risk factor for cardiovascular diseases but was also associated with cumulative organ damage [164]. Insulin resistance is highly prevalent in SLE, affecting approximately 40% of patients, and reflects multifactorial mechanisms—chronic inflammation, autoantibody-mediated effects, renal involvement (including nephrotic-range proteinuria), and glucocorticoid-related metabolic effects—that track with overall disease activity [165].

Atherosclerotic risk: The cardiovascular risk in SLE is among the highest of all autoimmune conditions. A systematic review and meta-analysis demonstrated that women aged 35–44 years with SLE exhibit dramatically elevated cardiovascular risk, with relative risks exceeding 2.5-fold for stroke and approaching 3-fold for myocardial infarction [18]. This elevated risk reflects the combined effects of traditional risk factors, disease-specific inflammatory mechanisms, and unique pathogenic features, including antiphospholipid antibodies and immune complex-mediated vascular injury. Regarding the role of metabolic modulators in cardiovascular complications of SLE, dysregulation of both innate and adaptive immune cells, along with their infiltration into kidney and vascular tissues, is a pivotal factor contributing to cardiovascular complications, with metabolic pathways in CD4+ T cells playing a central role [166]. Premature atherosclerosis is common, with studies demonstrating increased carotid intima-media thickness and coronary artery calcification in young SLE patients who would otherwise be considered low risk based on traditional risk assessment tools [167,168].

Thromboembolic risk: Certain clinical features of SLE and the presence of antiphospholipid antibodies (aPL) correlate with increased metabolic and cardiovascular risk [164]. Beyond traditional and inflammatory mechanisms, unique pathogenic features, including antiphospholipid antibodies and immune complex-mediated vascular injury, contribute independently to the markedly elevated cardiovascular risk observed in SLE, particularly among young women [18]. Interestingly, it was recently demonstrated that SLE-associated factors such as lupus anticoagulant and neurological SLE seem important for the onset of thromboembolic events. In addition, it was suggested that a higher CHA2DS2-VA score—a clinical score established by the European Society of Cardiology that stratifies thromboembolic risk in atrial fibrillation to guide oral anticoagulation decisions—could predict the occurrence of cardiovascular events in SLE patients [169]. These observations need to be confirmed by other studies, and validation of this score for predicting stroke risk is also awaited. Mechanistic pathways underlying these associations are summarized in Table A1; therapeutic and monitoring approaches are discussed in Cardiovascular Risk Management Strategies.

### 5.3. Antiphospholipid Syndrome

Antiphospholipid syndrome (APS), also known as Hughes syndrome, is a systemic autoimmune disorder characterized by the presence of antiphospholipid antibodies (aPL), including the three cardinal autoantibodies lupus anticoagulant (LA), anticardiolipin (aCL), and anti-beta2-glycoprotein I (anti-β2GPI), in association with arterial and/or venous thrombosis and obstetric complications. With an estimated incidence of 1–2 cases per 100,000 person years and a prevalence ranging from 40 to 50 cases per 100,000, APS can occur as a primary disorder or secondary to other autoimmune diseases, most commonly SLE [170]. Cardiovascular disease represents the foremost cause of morbidity and mortality in APS, driven by unique mechanisms involving both thrombo-inflammation and atherothrombosis that distinguish it from other autoimmune conditions [171].

Metabolic risk: MetS is highly prevalent in APS, though epidemiological data reveal interesting comparative patterns. A study comparing 138 APS patients with age- and sex-matched cohorts of rheumatoid arthritis and diabetes mellitus found that MetS prevalence was higher in APS (25.4–29.7%) than in RA patients (18.1–21.0%), but lower than in diabetes mellitus patients (48.6–53.6%) [172]. The same study revealed that APS patients with MetS had lower levels of physical activity, higher body mass index, and elevated cardiovascular risk biomarkers, including high-sensitive CRP, suggesting multiple pathways linking metabolic dysregulation to thrombotic and atherosclerotic complications [172]. Notably, while APS’s thrombo-inflammatory nature contributes to cardiovascular risk, it may also have implications for metabolic complications: chronic inflammation is a well-established driver of insulin resistance in other conditions, but a direct mechanistic link between APS-driven inflammation and insulin resistance has not been reported.

Atherosclerotic risk: Antiphospholipid antibodies directly promote endothelial dysfunction and atherosclerosis through multiple pathways [171]. Oxidized LDL (oxLDL) and anti-oxLDL antibodies have been implicated in vascular events underlying APS, creating a synergistic proatherogenic environment [173,174]. In APS, oxidized LDL (oxLDL) and anti-oxLDL antibodies do not merely coexist but form a pathogenic ternary complex with β2-glycoprotein I (β2GPI), one of the three principal autoantigens of the syndrome [175,176].

Thromboembolic risk: Within APS, MetS showed particularly strong associations with arterial thrombosis: patients with MetS exhibit significantly higher rates of arterial events and more extensive coronary atherosclerotic plaque burden on vascular ultrasound than those without [172]. Antiphospholipid antibody-mediated thrombo-inflammation represents an emerging pathogenetic mechanism of cardiovascular disease in APS, with these antibodies activating endothelial cells, increasing platelet adhesion, and influencing the balance of coagulation and anticoagulation molecules toward a prothrombotic state [171]. Mechanistic pathways underlying these associations are summarized in Table A1; therapeutic and monitoring approaches are discussed in Cardiovascular Risk Management Strategies.

Clinical management implications: Cardiovascular risk management in APS represents unique challenges (in addition to the general strategies below). A recent study examining cardiovascular risk factor management trends in APS versus RA and diabetes mellitus found that despite similar or higher cardiovascular risk, APS patients had lower rates of achieving therapeutic targets for blood pressure, LDL cholesterol, and other modifiable risk factors compared to both RA and diabetes cohorts [177], underscoring the need for heightened awareness and more aggressive cardiovascular risk modification in APS. The presence of MetS in APS is associated with increased cardiovascular events and vascular mortality, as well as development of chronic kidney disease and diabetes mellitus, emphasizing the importance of comprehensive metabolic screening and management [25]. Current recommendations include strict control of traditional risk factors, consideration of statin therapy even in the absence of hyperlipidemia (given its anti-inflammatory benefits), and careful attention to the metabolic effects of anticoagulation and immunosuppressive therapies used to manage the underlying condition.

### 5.4. Psoriasis and Psoriatic Arthritis

Psoriasis, affecting approximately 2–3% of the population, is increasingly recognized as a systemic inflammatory disease with metabolic consequences.

Metabolic risk: MetS prevalence ranges from 30% to 50% in psoriasis and rises with disease severity and body surface area involvement. The relationship appears bidirectional: obesity and metabolic dysfunction can exacerbate psoriasis, while chronic inflammation in psoriasis promotes metabolic dysregulation, with insulin resistance, dyslipidemia, hypertension, and abdominal obesity all more prevalent than in matched controls [178,179]. Obesity is also associated with increased risk of incident psoriatic arthritis among psoriasis patients, and weight reduction is recommended in psoriasis patients with MetS [180].

Atherosclerotic risk: Cardiovascular disease is a major driver of morbidity and mortality in psoriasis; risk increases with disease severity: meta-analyses show mild psoriasis carries modestly elevated risk of myocardial infarction, stroke, and cardiovascular death, rising further in psoriatic arthritis [181]. This risk is mechanistically linked to Th17-driven inflammation and production of IL-17, IL-23, and TNFα, which promote both cutaneous inflammation and vascular pathology. Directly supporting this link, FDG-PET/CT imaging studies show vascular inflammation and non-calcified coronary plaque burden correlating with psoriasis skin severity, independent of traditional cardiovascular risk factors [155].

Thromboembolic risk: A systematic review and meta-analysis of 13 cohort studies (over 12.4 million participants) reported in psoriasis patients an association with a significantly increased risk of incident VTE (pooled HR 1.26, 95% CI 1.08–1.48) and peripheral vascular disease (pooled HR 1.27, 95% CI 1.16–1.40). Subgroup analysis showed the elevated VTE risk was also present specifically among patients with psoriatic arthritis (pooled HR 1.24, 95% CI 1.01–1.53) and was more pronounced in women (pooled HR 1.89, 95% CI 1.36–2.61) [182].

### 5.5. Inflammatory Bowel Disease

Inflammatory bowel disease, encompassing Crohn’s disease (CD) and ulcerative colitis (UC), exhibits a distinct metabolic profile compared with other autoimmune conditions, driven by a paradoxical coexistence of inflammation-mediated and glucocorticoid-mediated insulin resistance alongside gut-specific metabolic abnormalities not prominently featured in other autoimmune conditions [183].

Metabolic risk: Although malnutrition and weight loss have traditionally dominated the metabolic paradigm in IBD, increased prevalence of MetS in this condition is now documented, with a higher burden in UC patients than CD patients that increases significantly with age [184,185]. Insulin resistance is significantly increased in hospitalized UC patients, as demonstrated by euglycaemic hyperinsulinemic clamp, and is associated with disease activity, glucocorticoid (GC) exposure, and systemic inflammation; the underlying study suggests that the combination of high inflammatory activity and GC treatment is more likely responsible for the insulin resistance during the acute phase of the disease [186]. The chronic inflammatory state, characterized by elevated TNFα, contributes to insulin resistance. A small prospective study reported that TNFα antagonism (infliximab/adalimumab) in nonobese, nondiabetic IBD patients significantly improved insulin resistance (reduced HOMA-IR index) independently of BMI or CRP, suggesting a causal contribution of TNFα to IBD-associated insulin resistance beyond disease activity alone [187]. Although IL-1β or IL-6 levels are elevated in IBD, no interventional studies targeting IL-1β or IL-6 with metabolic endpoints have been reported, and their contribution to IBD-associated insulin resistance is extrapolated from pathways established in obesity and obesity-related metabolic abnormalities rather than demonstrated directly. Gut dysbiosis, characteristic of IBD, marked by depletion of short-chain fatty acids (SCFA), producing Firmicutes and expansion of proinflammatory Proteobacteria, may also impair insulin sensitivity through reduced SCFA production, though direct clinical evidence in IBD patients remains limited [188,189].

Atherosclerotic risk: Cardiovascular disease risk is increased in IBD, although the relative risk appears modest in comparison to some other inflammatory autoimmune conditions. A meta-analysis of 12 studies reported that IBD patients had increased risk of acute coronary syndrome (ACS) in both adjusted (HR 1.23; 95% CI 1.08 to 1.41) and unadjusted analyses (HR 1.50; 95% CI 1.16 to 1.92), with important heterogeneity; the greater risk of ACS was observed in younger patients <40 years (relative HR 1.50; 95% CI 1.15 to 1.96) [190]. A second meta-analysis of 18 studies reviewed the incidence of heart failure and echocardiographic changes in IBD patients and revealed subtle yet significant structural and functional cardiac changes, including left ventricular and atrial dysfunction, vascular changes, arrhythmias, and heart failure hospitalization [191], findings that reflect a broader cardiac burden beyond atherosclerotic disease per se. The most recent meta-analysis of 23 studies confirmed that IBD patients are at higher risk of developing cardiovascular events during working age, with CD patients having a higher risk of cardiovascular disease, acute myocardial infarction, and stroke than UC patients [192]. This increased cardiovascular risk appears to track with disease activity, as during remission periods, the risk was comparable to that observed in control individuals [193]. Proposed contributing mechanisms include chronic systemic inflammation, endothelial dysfunction, and accelerated atherosclerosis [194]. Gut dysbiosis may further contribute through dysregulated bile acid signaling and elevated levels of the proatherogenic metabolite trimethylamine N-oxide (TMAO), although direct clinical evidence in IBD patients remains limited [188,189].

Thromboembolic risk: Thrombo-inflammatory activation has also been proposed as a contributing mechanism to the increased cardiovascular risk observed in IBD, alongside the chronic inflammatory and atherosclerotic pathways described above [194].

### 5.6. Autoinflammatory Syndromes

Autoinflammatory diseases—familial Mediterranean fever (FMF), TNF receptor-associated periodic syndrome (TRAPS), and cryopyrin-associated periodic syndromes (CAPS)—are monogenic disorders of innate immune dysregulation that share metabolic and cardiovascular features with polygenic autoimmune diseases, with subclinical inflammation often persisting between attacks [195].

Metabolic risk: Acute attacks are associated with insulin resistance and dyslipidemia [195].

Atherosclerotic risk: Acute attacks are also associated with endothelial dysfunction [195]. A systematic review and meta-analysis confirmed that FMF patients show increased carotid intima-media thickness and impaired flow-mediated dilation compared with controls, consistent with premature subclinical atherosclerosis [196]. AA amyloidosis, a complication of chronic inflammation in these diseases, further compounds cardiovascular risk via renal dysfunction [197,198]. Whether anti-inflammatory therapy (e.g., colchicine in FMF or IL-1 inhibition in CAPS) meaningfully reduces hard cardiovascular outcomes in autoinflammatory disease specifically remains unproven, and clinical trials for cardiovascular endpoints are awaited in this population.

Thromboembolic risk: For FMF specifically, a cross-sectional study of 6534 FMF patients and matched controls revealed that FMF was independently associated with venous thromboembolism after multivariate regression analysis adjusting for age, sex, socio-economic status, and VTE-associated comorbidities (HR 1.96, *p* < 0.001), likely reflecting the chronic inflammatory state of the disease [199]. No such association has been reported in CAPS or TRAPS to date.

## 6. Immunometabolic Reprogramming in Autoimmune Diseases

Immunometabolism describes the bidirectional relationship between intracellular metabolic pathways and immune cell fate and function, which reoriented cellular metabolism from a passive energy supplier to an active determinant of immune responses [200]. Immune cells undergo precise metabolic reprogramming during quiescence, activation, differentiation, and memory formation to meet specific bioenergetic and biosynthetic demands. For instance, activated macrophages and T-cells rely on glycolysis for their function, whereas memory T cells and regulatory T cells (Treg) preferentially utilize oxidative phosphorylation (OXPHOS) for their energetic needs [201,202]. In autoimmune diseases, this regulation becomes pathologically disrupted: metabolic reprogramming links cellular energy needs to immune dysfunction, driving inflammation, tissue damage, and aberrant immune cell differentiation, proliferation, and cytokine secretion [203,204]. The resulting immunometabolic landscape, characterized by a glycolytic Warburg-like shift, mTOR/HIF-1α activation, mitochondrial dysfunction, M1 macrophage polarization, and constitutive NF-κB signaling, forms a self-reinforcing network that maintains chronic autoimmune activation and drives disease progression [205,206,207] (Figure 1). The clinical relevance of this framework is validated by the mechanism of action of established immunomodulatory drugs. Rapamycin (sirolimus, Rapamune) inhibits mTORC1, thereby suppressing Th17 cell differentiation through mTORC1-HIF1α-dependent inhibition of glycolytic reprogramming while expanding Tregs, a metabolic divergence due to the fact that Tregs for their immune suppressive function rely more on OXPHOS fuelled by FA oxidation [208,209]. Patients enrolled in a single-arm, open-label study of clinically active SLE resistant or intolerant to conventional therapy, who were treated with rapamycin for 12 months, demonstrated a progressive improvement in disease activity that was associated with correction of proinflammatory T-cell lineage specification [210]. Besides rapamycin, dimethylfumarate (DMF), metformin, and methotrexate (MTX) also target immunometabolism by regulating or mimicking endogenous metabolites that limit or block inflammation [211]. For instance, DMF, approved for relapsing-remitting multiple sclerosis, mimics endogenous TCA-cycle immunometabolites itaconate and fumarate to drive antioxidant gene expression, reduce proinflammatory cytokine production, and repolarize macrophages toward an anti-inflammatory phenotype [211]. As for MTX, the first-line treatment agent in RA, psoriatic arthritis, and other forms of inflammatory arthritis, multiple mechanisms have been proposed to explain its anti-inflammatory action, including the inhibition of purine and pyrimidine synthesis, transmethylation reactions, translocation of NF-κB to the nucleus, signaling via the JAK-STAT pathway and nitric oxide production, as well as the promotion of adenosine release [211,212].

Altogether, these observations establish immunometabolic reprogramming as a proactive biological driver in the pathogenesis of autoimmune diseases. Already existing approved agents act by rewiring immune cell metabolism to improve disease activity progression. Thus, the quest for new molecules targeting immunometabolic processes will help improve therapeutic care in patients with autoimmune conditions that are resistant or intolerant to approved medicines.

## 7. Therapeutic Agents and Their Metabolic Consequences

### 7.1. Glucocorticoids: Diabetogenic Effects and Management

Glucocorticoids remain among the most widely prescribed anti-inflammatory agents in autoimmune diseases, but their metabolic cost is under-recognized: over 10% of United Kingdom inpatients receive them, and they account for roughly 2% of new-onset diabetes globally, yet glucose monitoring is inconsistent [213]. Glucocorticoids drive hyperglycemia through effects at multiple sites. In peripheral tissues, they impair insulin signaling, inducing inhibitory IRS1 serine phosphorylation, activating protein tyrosine phosphatase 1B (which dephosphorylates and inactivates the insulin receptor), and upregulating tribbles homolog 3 (TRB3) to block Akt phosphorylation, while reducing GLUT4 translocation to the cell membrane and glycogen synthase activity in skeletal muscle. In liver, they upregulate PEPCK and glucose-6-phosphatase (raising gluconeogenesis) and promote steatosis via sterol regulatory element-binding protein-1c (SREBP-1c)-driven lipolysis and adipose tissue-derived free fatty acid delivery. In pancreatic β-cells, they impair glucose sensing (via GLUT2/glucokinase downregulation) and blunt insulin exocytosis through potassium channel-mediated hyperpolarization, producing a characteristic pattern of high basal but blunted glucose-stimulated insulin secretion. In adipose tissue, they promote visceral fat accumulation and lipolysis, amplified locally by 11β-hydroxysteroid dehydrogenase type 1 (11βHSD1)-mediated cortisone-to-cortisol conversion [214,215], which impairs further insulin sensitivity, supporting the “dehydrogenase hypothesis” of tissue-specific glucocorticoid excess contributing to MetS [216].

Clinically, glucocorticoid-induced hyperglycemia is predominantly postprandial, so fasting glucose and HbA1c can both underestimate true glycemic exposure; postprandial monitoring is preferred [217].

For prevention and treatment, metformin was shown in a randomized, placebo-controlled, proof-of-concept trial in patients on long-term glucocorticoid therapy to improve glucose and lipid parameters and reduce inflammatory markers and ameliorate fibrinolysis, as well as carotid intima-media thickness, warranting further investigation in high-risk patients starting glucocorticoids [218]. Cardiovascular risk with glucocorticoid is time- and dose-dependent but present even at low doses; registry data show increased cardiovascular events with prednisone doses ≥5 mg/day [219,220], reinforcing current guidance favoring glucocorticoid-sparing DMARDs/biologic strategies, with glucocorticoids used at the lowest effective dose for the shortest duration when unavoidable.

### 7.2. Disease-Modifying Antirheumatic Drugs (DMARDs) and Metabolic Effects

Effective control of inflammatory disease activity may help mitigate metabolic complications in autoimmune conditions, though the causal contribution of specific DMARDs remains inconsistently defined. In a cohort of RA patients, methotrexate use was associated with a 24% reduction in composite cardiovascular events and a 57% reduction in heart failure hospitalisations, though notably, this benefit was not mediated through reduced RA disease activity, suggesting mechanisms beyond simple anti-inflammatory action [221]. This contrasts with the CIRT trial, in which low-dose methotrexate (MTX) failed to reduce inflammatory markers or cardiovascular events in patients with atherosclerosis without RA [222], underscoring that MTX’s cardiometabolic effects in RA may be disease-context-specific rather than a generalizable anti-inflammatory mechanism. A single cross-sectional study also reported reduced prevalence of MetS with MTX use in RA patients over 60 [223], though this association requires confirmation in prospective, causally designed studies. TNFα inhibitors, including infliximab, adalimumab, etanercept, golimumab, and certolizumab, have transformed treatment of RA, psoriasis, psoriatic arthritis, and IBD, with evidence of metabolic benefit beyond disease control. A meta-analysis of 10 longitudinal studies (297 patients) found anti-TNFα therapy significantly reduced insulin resistance and improves insulin sensitivity (HOMA-IR, QUICKI) in active RA [224]. Similar insulin-sensitizing effects have been reported with infliximab/adalimumab in nonobese, nondiabetic IBD patients [187], and with adalimumab in moderate-to-severe psoriasis, suggesting the effect extends across TNF-driven inflammatory conditions rather than being RA-specific [24].

However, TNFα inhibitors typically raise both HDL-C and LDL-C levels, partially reversing the “lipid paradox” of inflammatory disease, in which suppressed cholesterol during active RA paradoxically associates with higher cardiovascular risk rather than lower risk [83]. This rise in LDL-C should not be read in isolation: it occurs alongside improved HDL function and reduced inflammation, which was confirmed by meta-analyses [225], consistent with a net neutral-to-favorable cardiovascular effect rather than a straightforward atherogenic signal. IL-6 blockade with tocilizumab has significant metabolic implications. Because sustained IL-6 signaling promotes hepatic insulin resistance and alters lipoprotein metabolism, IL-6 inhibition might be expected to improve some metabolic parameters, while paradoxically worsening lipid profiles [226,227]. Indeed, small clinical studies have shown improved insulin sensitivity with tocilizumab in RA patients [31,32]. Paradoxically, however, tocilizumab significantly raises LDL-C and triglycerides, often requiring initiation or intensification of lipid-lowering therapy. Despite this adverse lipid profile, the ENTRACTE, a randomized trial comparing it with etanercept in over 3000 patients with RA and cardiovascular risk factors, found no excess cardiovascular risk with tocilizumab, suggesting its anti-inflammatory effects offset the lipid changes, yet other adverse effects, including serious infection and gastro-intestinal perforation, occurred more frequently in the tocilizumab group [228]. IL-1 inhibition with anakinra, canakinumab, or rilonacept has shown efficacy in monogenic autoinflammatory syndromes, with emerging interest in selected autoimmune conditions. The landmark CANTOS trial (Canakinumab Anti-inflammatory Thrombosis Outcomes Study) demonstrated that canakinumab reduced recurrent cardiovascular events in patients with prior myocardial infarction and elevated CRP, independent of lipid lowering, establishing proof-of-concept for the inflammatory hypothesis of atherothrombosis [60]. Separately, in patients with type 2 diabetes, IL-1 receptor antagonism with anakinra improved glycemia and beta-cell secretory function and reduced inflammatory markers, though insulin sensitivity itself was unchanged [229]. In autoimmune diseases such as rheumatoid arthritis, IL-1 blockade with anakinra not only controls disease activity but has also been shown, in a randomized trial of patients with comorbid type 2 diabetes, to improve glycemic control and insulin resistance, outperforming TNF inhibition in this setting [46]. These findings have important implications for understanding the inflammation–metabolism interface to help identify novel therapeutic targets.

Other targeted therapies carry distinct metabolic and cardiovascular considerations. JAK inhibitors (tofacitinib, baricitinib, upadacitinib) raise LDL-C and HDL-C, similar to IL-6 inhibitors, but more importantly, the FDA-mandated ORAL Surveillance trial found tofacitinib associated with higher rates of major adverse cardiovascular events and cancer than TNFα inhibitors, prompting warnings across the class [230]. Among other options, abatacept has shown improved insulin sensitivity in observational studies [231], while comparable metabolic data for rituximab are lacking. Overall, effective inflammatory control often coincides with improved metabolic parameters, but this relationship is not universal, as ORAL Surveillance demonstrates; anti-inflammatory efficacy does not guarantee improved cardiovascular outcomes, and agent-specific risk profiles require individualized monitoring.

### 7.3. Metabolic Syndrome and Response to Advanced Therapies

An important, clinically relevant observation is that MetS may influence response to advanced therapies in autoimmune diseases. A recent study of RA patients with MetS initiating TNF inhibitor or non-TNF biologic therapy found that MetS was associated with significantly lower odds of achieving the minimal clinically important difference (MCID) in Clinical Disease Activity Index (CDAI) at 6 months, with similar effects across TNF inhibitor and non-TNF therapy groups; the magnitude of reduced response correlated with the number of MetS components present, suggesting a dose–response relationship [103].

These findings carry three clinical implications: addressing metabolic abnormalities before or alongside advanced therapy initiation may improve outcomes; patients with MetS may need more intensive or alternative treatment strategies; and comprehensive metabolic assessment should be seen as integral to disease management, directly influencing treatment efficacy rather than as separate comorbidity care. Mechanistically, this reduced response likely reflects adipose-tissue-derived proinflammatory cytokine production overwhelming cytokine-targeted therapies, consistent with observations showing that obesity reduces the effectiveness of cytokine-targeted (but not cell-targeted) biologics in RA [232].

## 8. Cardiovascular Risk Management Strategies

### 8.1. Risk Assessment and Stratification

Comprehensive cardiovascular risk management in patients with autoimmune and autoinflammatory diseases requires integrating traditional risk factor modification with disease-specific considerations. Standard cardiovascular risk calculators, such as the Framingham Risk Score or Pooled Cohort Equations, substantially underestimate cardiovascular risk in autoimmune disease populations, as they do not account for chronic inflammation and disease-specific factors. In one RA cohort, observed cardiovascular event rates were roughly double those predicted by the Framingham score in women and 65% higher in men [233]. Accordingly, the European League Against Rheumatism (EULAR) recommends applying a 1.5-fold multiplication factor to calculated cardiovascular risk scores in RA and other inflammatory joint diseases, which can shift more patients into higher standard-guideline risk categories, though optimal disease-specific risk stratification remains an area of active investigation [234]. Novel biomarkers may improve risk stratification beyond traditional factors. Inflammatory markers, including high-sensitivity CRP, IL-6, and TNFα, correlate with cardiovascular events in autoimmune diseases, though their incremental predictive value beyond established risk scores requires further validation. Disease activity measures specific to each condition (e.g., DAS28 in RA, SLEDAI in SLE) predict cardiovascular events and should be incorporated into risk assessment. Imaging modalities, including carotid ultrasound for intima-media thickness, coronary artery calcium scoring, and arterial FDG-PET, can detect subclinical atherosclerosis and may help identify high-risk individuals who would benefit from more intensive preventive interventions.

### 8.2. Lipid-Lowering Therapy and Anti-Inflammatory Benefits

Lipid-lowering therapy with statins represents a cornerstone of cardiovascular prevention in high-risk populations. In addition to lowering LDL cholesterol, statins have pleiotropic effects and may exert anti-inflammatory, antioxidant, antithrombotic, immunomodulatory, and endothelial-protective actions. These actions include reduced oxidative stress, improved nitric oxide bioavailability, decreased endothelial adhesion-molecule expression, attenuation of vascular inflammation, and modulation of platelet and immune-cell activation [235]. Meta-analyses in RA support both efficacy and anti-inflammatory benefit, reducing CRP, erythrocyte sedimentation rate, and disease activity, beyond their lipid-lowering effect [236]. Accordingly, current European (EULAR) guidelines recommend a higher risk-score multiplier in inflammatory joint diseases than in the general population [234], while ACC/AHA guidelines list chronic inflammatory disease as a parallel risk-enhancing factor [237]. A key consideration is the “lipid paradox”: during active inflammation, traditionally favorable (low) cholesterol levels can paradoxically coincide with higher cardiovascular risk, so lipids should ideally be assessed once disease activity is stable [83]. Rather than constituting an independent, disease-specific indication, high disease activity, longer disease duration, or other inflammatory features should be incorporated as risk-modifying factors into individualized cardiovascular risk assessment performed within existing prevention guideline frameworks (e.g., the EULAR risk-score multiplier [234]), with statin initiation following standard guideline-based risk thresholds even in patients without overt hyperlipidemia. Combination lipid-lowering strategies (e.g., ezetimibe, PCSK9 inhibitors) may be needed for persistent dyslipidemia, particularly with familial hypercholesterolemia or established cardiovascular disease.

### 8.3. Management of Diabetes and Insulin Resistance

Management of diabetes and insulin resistance in autoimmune disease patients requires attention to both glycemic control and the broader metabolic context. Metformin, the first-line agent for type 2 diabetes, offers potential additional benefits in autoimmune diseases beyond glucose lowering, including anti-inflammatory effects and possible disease-modifying properties. A randomized controlled trial in RA patients found that adding metformin to conventional synthetic DMARDs significantly reduced CRP and disease activity (DAS28-CRP) compared with placebo, with cardiovascular benefit proposed as a plausible but not yet directly demonstrated additional outcome [238]. Another randomized placebo-controlled trial reported improved DAS-28 and other inflammatory cytokines with metformin + MTX in RA patients compared to MTX-treated patients [239]. Separately, a randomized, placebo-controlled phase 2 trial showed that metformin reduced metabolic complications and inflammatory markers in patients on chronic systemic glucocorticoid therapy, supporting its use in patients requiring long-term glucocorticoids [218].

Newer antidiabetic agents with proven cardiovascular benefits represent attractive options for patients with autoimmune diseases and diabetes or high cardiovascular risk. GLP-1 receptor agonists (GLP1-RA) liraglutide, semaglutide, and dulaglutide have demonstrated cardiovascular risk reduction in large outcomes trials (LEADER and SUSTAIN-6 trials) and offer additional benefits of weight loss and potential anti-inflammatory effects [240,241]. SGLT2 inhibitors (empagliflozin, dapagliflozin, canagliflozin) provide cardiovascular and renal protection as shown in EMPA-REG OUTCOME [242], CANVAS/CREDENSE [243], and DECLARE-TIMI 58 trials [244]. While specific outcome data in autoimmune disease populations are still emerging, the mechanistic rationale and general population data support consideration of these agents in appropriate patients. For instance, RA patients treated with GLP1-RA (semaglutide or tirzepatide) experienced significantly greater reductions in RA disease activity, pain, body weight, and other metabolic abnormalities compared with RA patients who were prescribed but did not take GLP1-RA [245]. These findings suggest a potential benefit of GLP-1RAs in improving RA disease activity and cardiovascular risk profile in this population. Similarly, a retrospective study reported improved disease activity in SLE patients on GLP-1RAs, alongside favorable weight/metabolic outcomes [246]. These observations need to be confirmed in large prospective randomized placebo-controlled studies in RA and SLE patients but also extended to target disease activity and metabolic complications in other autoimmune diseases in appropriate patients.

### 8.4. Lifestyle Interventions and Multidisciplinary Care

Lifestyle modifications, including diet, physical activity, smoking cessation, and weight management, remain fundamental components of metabolic and cardiovascular risk reduction. However, implementation is constrained by disease-specific barriers: pain, fatigue, and functional limitations are consistently reported as the dominant obstacles to physical activity in RA and related conditions, mediated in part by proinflammatory cytokines such as IL-6 acting on the hypothalamic-pituitary-adrenal (HPA) axis and central nervous system (CNS) [247]. Additionally, glucocorticoid therapy independently contributes to weight gain, central adiposity, and insulin resistance [248], compounding the effects of disease-associated physical inactivity. Despite these challenges, structured exercise programs tailored to individual capabilities have demonstrated benefits in multiple autoimmune conditions, improving not only cardiovascular fitness and metabolic parameters but also reducing disease activity and improving quality of life [249].

Dietary interventions represent an area of growing interest, with particular attention to anti-inflammatory dietary patterns such as the Mediterranean diet. This dietary pattern, characterized by high consumption of fruits, vegetables, whole grains, legumes, nuts, and olive oil, with moderate fish intake and limited red meat and processed foods, has demonstrated cardiovascular benefits in the general population [250,251] and shows promise in autoimmune diseases. Studies in RA patients have reported improvements in disease activity, inflammatory markers, and physical function with Mediterranean diet adherence [252,253]. The potential mechanisms involve not only the favorable effects on traditional cardiovascular risk factors but also direct anti-inflammatory properties of specific nutrients and bioactive compounds.

Multidisciplinary care coordination, involving rheumatologists, cardiologists, endocrinologists, dietitians, and other healthcare professionals, is essential for comprehensive management of metabolic complications in autoimmune diseases. Systematic screening for cardiovascular risk factors, MetS components, and subclinical cardiovascular disease should be integrated into routine care. Patient education regarding the increased cardiovascular risk associated with their inflammatory disease and the importance of both disease control and traditional risk factor management is crucial for promoting engagement with preventive strategies and long-term adherence to therapeutic interventions.

### 8.5. Future Directions and Emerging Therapies

Immunometabolism, the study of bidirectional interactions between immune activation and metabolic pathways, is a rapidly evolving field with potential to yield therapies that simultaneously address inflammatory disease activity and metabolic dysfunction, for example, through inhibitors of glycolysis or glutaminolysis in activated T cells [254].

Advanced multi-omics phenotyping is beginning to reveal disease subsets with distinct metabolic profiles, supporting precision-medicine approaches that integrate inflammatory and metabolic biomarkers for personalized treatment selection and cardiovascular risk stratification. Disease-specific cardiovascular risk models incorporating inflammatory markers and therapy exposure would further improve risk assessment.

Emerging therapeutic targets include adipose tissue macrophage recruitment, adipokine secretion, adiponectin enhancement, and HDL function, offering complementary approaches to existing anti-inflammatory therapies. The gut microbiome has also emerged as a key modulator of both immune function and metabolism, as well as microbiome-targeted interventions, including probiotics, prebiotics, and fecal microbiota transplantation, warranting investigation for their potential to jointly improve inflammatory disease control and metabolic health [255].

Selective glucocorticoid receptor modulators (SEGRMs) that dissociate anti-inflammatory from adverse effects on glucose and bone metabolism remain an active but challenging area of drug development [256]. Better understanding of tissue-specific glucocorticoid action, particularly the role of 11βHSD1 in amplifying local glucocorticoid effects, may enable tissue-selective inhibitors that preserve anti-inflammatory benefit while minimizing metabolic harm.

## 9. Conclusions

Metabolic complications are a significant, underrecognized driver of excess cardiovascular morbidity and mortality in autoimmune and autoinflammatory diseases, with MetS affecting ~30% of patients and cardiovascular risk elevated 1.5- to 3-fold versus the general population. These complications arise from cytokine-driven disruption of metabolic signaling, altered adipokine profiles, endothelial dysfunction, and proatherogenic immune activity, making them integral disease features rather than incidental comorbidities.

Clinical management of these patients requires simultaneously controlling inflammation and metabolic risk. Effective DMARDs/biologic therapy improves both disease activity and metabolic parameters, while glucocorticoids, though often necessary, drive insulin resistance, dyslipidemia, and central obesity, warranting glucocorticoid-sparing strategies. Metformin may be considered individually in patients with documented hyperglycemia, prediabetes, or particularly high metabolic risk, rather than used routinely for prophylaxis in all glucocorticoid-treated patients. MetS may also blunt treatment response, reinforcing the need for metabolic assessment as part of therapeutic optimization.

Cardiovascular risk reduction should be guided by statin eligibility criteria established from cardiovascular-prevention guidelines, recognizing that conventional risk calculators may underestimate risk in chronic inflammatory disease. Additional measures include diabetes agents with proven cardiovascular benefit, disease-adapted lifestyle interventions, and inflammation-aware risk stratification tools.

Future work should clarify the molecular links between inflammatory pathways and metabolic dysfunction, advance precision-medicine risk stratification, and explore therapies targeting the immunometabolic interface, ultimately aiming to reduce cardiovascular burden and improve long-term outcomes in these patients.

## Data Availability

No new data were created or analyzed in this study. Data sharing is not applicable to this article.

## Appendix A

**Table A1:** Main cytokines involved in metabolic complications of autoimmune and autoinflammatory diseases.

Cytokine/Mediator	Metabolic Complication	Disease Association	References
TNFα	Insulin resistance	RA, Psoriasis, PsA	[20,23,24]
	Dyslipidemia/CVD	SLE	[21,22]
	Venous thrombosis	APS	[26]
IL-6	Insulin resistance	RA	[30,31,32]
	Dysfunctional proinflammatory HDL/CVD	RA, SLE	[34]
	Thrombo-inflammatory vasculopathy	APS	[26]
IL-1β	Insulin resistance	RA; RA with comorbid T2D	[45,46]
	CVD risk	CAPS	[60]
IL-18	Endothelial cell differentiation; insulin resistance; TG, hyperhomocysteinemia, vascular stiffness/CVD risk	SLE	[48,49,50]
	CVD risk (correlation with cIMT but not with HOMA-IR)	RA	[51]
NLRP3 Inflammasome	MetS/CVD risk	APS, RA	[55]
	Immunometabolism dysregulation in NLRP3 variants	CAPS	[59]
IL-17A/IL-23 axis	CVD risk	Psoriasis	[65]
	Improved metabolic parameters (leptin, cholesterol, TG, HDL-C) and glucose/inflammatory markers with anti-IL-17/IL-23 therapy	Psoriasis	[73,74]
Interferons	Dyslipidemia and impaired β-cell function (IFN-α and IFN-γ)	SLE	[75]
	Mitochondrial dysfunction, impaired metabolic fitness of CD8+ T cells (IFN-α)	SLE	[76]
	Subclinical vascular damage and cardiovascular markers (IFN-α)	SLE	[77]
	Impaired endothelial progenitor function (IFN-α)	APS	[78]
MCP-1/CCL2	Elevated MCP-1 directly associated with insulin resistance status	RA	[36]
Leptin	Insulin resistance (direct association independent of BMI and inflammatory markers)	RA	[108,136]
	Insulin resistance, TG, BMI	SLE	[138]
	Carotid artery intima-media thickness	Psoriasis	[140]
	Insulin resistance	APS	[25]
Adiponectin (paradox/deficit)	Increased levels correlate with disease severity, radiographic progression, and CVD risk	RA, SLE, PsA (paradox)	[106,115,126]
	Decreased levels correlate with BMI	Psoriasis + MetS (true deficit)	[121,122]
	Negative correlation with metabolic parameters (HOMA-IR, cholesterol, TG, visfatin)	RA (untreated, early diagnosed)	[126]
Resistin	Positive correlation with mean intima-media thickness/CVD risk	Psoriasis	[140]
	Positive correlation with cholesterol and triglycerides, and association with T2D and MetS	Psoriasis	[141]
Visfatin/NAMPT	HOMA-IR elevation, dyslipidaemia	RA (strongest in early untreated disease)	[126,128]

Abbreviations: RA, rheumatoid arthritis; PsA, psoriatic arthritis; APS, antiphospholipid syndrome; SLE, systemic lupus erythematosus; CAPS, Cryopyrin-Associated Periodic Syndromes; CVD, cardiovascular disease; MetS, metabolic syndrome; T2D, type 2 diabetes; HOMA-IR, homeostatic model assessment of insulin resistance; NAMPT, nicotinamide phosphoribosyltransferase; NLRP3, NOD-like receptor protein 3; BMI, body mass index; TG, triglycerides; cIMT: carotid intima-media thickness.
